# Harnessing novel chromosomal integration loci to utilize an organosolv‐derived hemicellulose fraction for isobutanol production with engineered *Corynebacterium glutamicum*

**DOI:** 10.1111/1751-7915.12879

**Published:** 2017-11-08

**Authors:** Julian Lange, Felix Müller, Ralf Takors, Bastian Blombach

**Affiliations:** ^1^ Institute of Biochemical Engineering University of Stuttgart D‐70569 Stuttgart Germany

## Abstract

A successful bioeconomy depends on the manifestation of biorefineries that entirely convert renewable resources to valuable products and energies. Here, the poorly exploited hemicellulose fraction (HF) from beech wood organosolv processing was applied for isobutanol production with *Corynebacterium glutamicum*. To enable growth of *C. glutamicum* on HF, we integrated genes required for d‐xylose and l‐arabinose metabolization into two of 16 systematically identified and novel chromosomal integration loci. Under aerobic conditions, this engineered strain CArXy reached growth rates up to 0.34 ± 0.02 h^−1^ on HF. Based on CArXy, we developed the isobutanol producer strain CIsArXy, which additionally (over)expresses genes of the native l‐valine biosynthetic and the heterologous Ehrlich pathway. CIsArXy produced 7.2 ± 0.2 mM (0.53 ± 0.02 g L^−1^) isobutanol on HF at a carbon molar yield of 0.31 ± 0.02 C‐mol isobutanol per C‐mol substrate (d‐xylose + l‐arabinose) in an anaerobic zero‐growth production process.

## Introduction

The future shortage of fossil oil and energy resources raises the demand for a sustainable bioeconomy which mitigates greenhouse gas emissions, relies on alternative energies and exploits renewable material streams and value chains. Biorefineries play a key role in processing lignocellulosic materials (reviewed in Cherubini, 2010; Valdivia *et al*., 2016; Rabaçal *et al*., 2017) but require a high efficiency in holistically converting the input biomasses in an economic manner to marketable products and energies. Because of the complexity and variability of the lignocellulosic feed, side streams evoke from conversion technologies such as the organosolv processing or fast pyrolysis, which are tedious to exploit and therefore limit the overall efficiency of the applied biorefinery approach. With respect to their abundance, hemicelluloses, which constitute between 15% and 35% of lignocellulosic biomass (Sauer *et al*., 2014), have initiated much consideration for biotechnological applications (Álvarez *et al*., 2016). However, they are still commonly wasted (Gírio *et al*., 2010) due to their complexity, limited accessibility for microorganisms and potential to form toxic components (e.g. weak acids and furan derivatives).

During the organosolv processing, a mixture of lignocellulose, organic solvent (e.g. ethanol), water and catalysts (e.g. sulfuric acid) is heated to 180–210°C, which fractionizes fibres (cellulosic material) and a black liquor (containing lignin and hemicelluloses; reviewed in Brosse *et al*., 2017; Zhao *et al*., 2017). After recovery of the organic solvent by distillation, the black liquor is diluted with water to yield precipitated lignin and the remainder liquid HF (Zhao *et al*., 2009). Cellulosic fibres can be enzymatically saccharified and used for fermentation purposes (Zhao *et al*., 2017) and high purity lignin fractions for example for functionalized materials, fuels, biodegradable polymers or adhesives (Brosse *et al*., 2011; Liu *et al*., 2015). Typically, the HF comprises weak acids, sugars (e.g. d‐xylose, l‐arabinose, d‐glucose, d‐mannose, d‐galactose), furan derivatives, phenolic residues and other extractives, and was proposed to be used for fermentation and production of chemicals (e.g. xylitol, furfural) (Zhao *et al*., 2009). Still, due to its complexity, the HF remains difficult to access. The need for technologies that utilize the HF without further laborious treatments lies therefore at hand. Microorganisms generally possess a versatile metabolism allowing in principle the conversion of such complex substrate mixtures to value‐added products through fermentation processes.

In this study, we applied the industrial workhorse *Corynebacterium glutamicum*, which has a long tradition in biotechnological production of amino acids but is also exploited for the biosynthesis of organic acids, alcohols and specialty chemicals (Liebl, 2005; Becker and Wittmann, 2015). This Gram‐positive, facultatively anaerobic bacterium (Nishimura *et al*., 2007) is robust and accepted as suitable candidate for future biorefinery applications (Jojima *et al*., 2013). Previously, *C. glutamicum* has been engineered to produce isobutanol, a next‐generation biofuel and precursor for chemical synthesis of rubber and specialty chemicals, from glucose (Smith *et al*., 2010; Blombach *et al*., 2011; Yamamoto *et al*., 2013). Alternative carbon source utilization has been implemented in tailored strains (Leßmeier *et al*., 2015) and harnessed for production of e.g. l‐lysine from pretreated hemicellulosic materials (Gopinath *et al*., 2011). However, hemicelluloses such as the organosolv‐derived HF have not been assayed for isobutanol production so far. Although tools for genetic engineering, omics and systems level analysis of this industrial workhorse are available (Kirchner and Tauch, 2003; Eggeling and Bott, 2005; Wendisch *et al*., 2006; Burkovski, 2015; Cho *et al*., 2017; Lee and Wendisch, 2017), there is still a need for suitable chromosomal sites to integrate genetic information, such as synthetic operons, to expand the metabolism for enhanced substrate consumption or production purposes. This issue was the moving cause to systematically identify suitable gene integration loci in this study. We inserted synthetic operons for d‐xylose and l‐arabinose metabolization into two of these sites to enable aerobic growth and anaerobic isobutanol production on HF with engineered *C. glutamicum* strains.

## Results and discussion

### Identification of *Corynebacterium glutamicum* landing pads (CgLPs)

**Figure 1:**
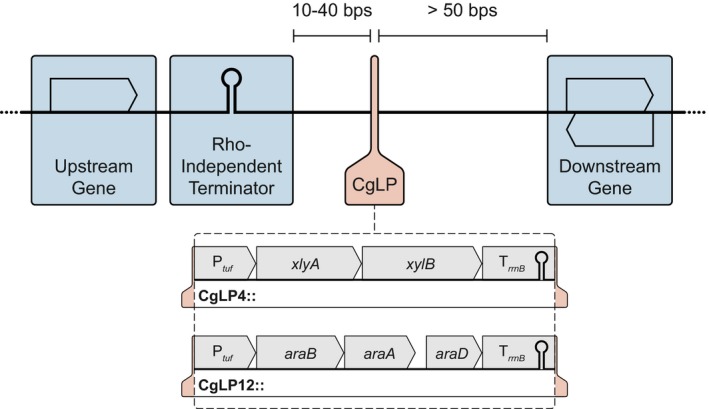
Schematic chromosomal location of *C. glutamicum* landing pads (CgLP) for chromosomal integration of genetic information. The synthetic operons P_*tuf*_‐*xylAB*‐T_*rrnB*_ and P_*tuf*_‐*araBAD*‐T_*rrnB*_ for d‐xylose and l‐arabinose metabolization were inserted exemplarily into CgLP4 and CgLP12 respectively. P_*tuf*_: promoter of the *C. glutamicum* elongation factor EF‐TU (cg0587); T_*rrnB*_: terminator of the *E. coli rrnB* operon; *xylAB*: genes encoding XylA (xylose isomerase) of *Xanthomonas campestris* and XylB (xylulokinase) of *C. glutamicum*;* araBAD*: encoding AraB (l‐ribulokinase), AraA (l‐arabinose isomerase) and AraD (l‐ribulose‐5‐phosphate 4‐epimerase) of *E. coli* MG1655. Arrows indicate gene direction.

Metabolic engineering aims at enhancing the substrate or product spectrum of microorganisms, which is a crucial prerequisite to fully exploit their biotechnological potential. This essentially requires the integration of additional genetic information into the host chromosome to circumvent the inherent disadvantages of plasmid‐based gene expression. So far, no general strategy to identify suitable spots for insertion was formulated. To propose such gene integration loci (designated as *C. glutamicum* landing pads, CgLPs), we harnessed the knowledge about transcription units (Pfeifer‐Sancar *et al*., 2013), non‐essential gene clusters (Unthan *et al*., 2014) and prophage regions (Kalinowski, 2005). First, the three prophage regions of *C. glutamicum* [CGP1 (cg1507‐cg1524), CGP2 (cg1746‐cg1752) and CGP3 (cg1890‐cg2071)] were excluded from the search for relevant integration sites (Kalinowski, 2005). Although they were shown to be non‐relevant for ordinary growth under laboratory conditions, the overall function is to date not clarified in depth and a genetic stability is not guaranteed (Baumgart *et al*., 2013). Second, we contemplated non‐essential chromosome sections in the published list of deletable regions (Unthan *et al*., 2014). These provide ideal arrays for the integration of genes and exclude lethal effects that arise from disruption of essential genetic structures. Third, the non‐essential regions were analysed for suitability regarding knowledge about transcription start sites, operon structures and Rho‐independent termination sites (Pfeifer‐Sancar *et al*., 2013). In total, 16 landing pads were identified throughout the chromosome as suitable spots for integration of additional genetic information (cf. Table 1, Fig. S1). All CgLPs locate after a Rho‐independent terminator of the upstream gene and are succeeded by a downstream gene stop or start codon at > 50 bps spacing (Fig. 1, Table 1). The distance between the CgLP and the upstream gene terminator was chosen between 10 and 40 bps depending on the size of the intergenic region. Integration of synthetic gene constructs should in general provide a strong termination site to minimize downstream effects. Two of the identified integration loci, CgLP4 and CgLP12, were exemplarily used in this study for integration of synthetic operons for d‐xylose and l‐arabinose metabolization respectively (cf. Fig. 1, Table 1).

**Table 1:** Compilation of identified *C. glutamicum* landing pads (CgLPs) for chromosomal integration of additional genetic information

*C. glutamicum* Landing Pad	Base Position^a^	Adjacence^b^	Spacer^c^	Upstream gene^d^	Downstream gene/operon^e^	Experimental verification
CgLP1	97220	◁ **⌇** ⋂ ◄	20	cg0121	cg0120	–
CgLP2	287966	► ⋂ **⌇** ◁	20	cg0327	cg0328	–
CgLP3^f^	558101	► ⋂ **⌇** ◁	40	cg0634 (*rplO*)^g^	cg0635	–
CgLP4	836158	► ⋂ **⌇** ▷	10	cg0901	cg0902	*xylAB*
CgLP5	837445	► ⋂ **⌇** ▷	20	cg0903	cg0904	–
CgLP6^f^	857008	► ⋂ **⌇** ▷	20	cg0928^g^	*rrnB*	–
CgLP7	1205320	◁ **⌇** ⋂ ◄	20	cg1302	cg1301 (*cydA*)	–
CgLP8	1427460	► ⋂ **⌇** ▷	40	cg1538 (*coaE*)^g^	cg1540	–
CgLP9	2741407	► ⋂ **⌇** ◁	40	cg2880	cg2883	–
CgLP10^f^	2971748	◁ **⌇** ⋂ ◄	40	cg3112 (*cysZ*)^g^	cg3111	–
CgLP11	3077633	► ⋂ **⌇** ▷	10	cg3212	cg3213	yes^h^
CgLP12	3094266	► ⋂ **⌇** ▷	20	cg3227 (*lldD*)	cg3228	*araBAD*
CgLP13	3191992	► ⋂ **⌇** ▷	10	cg3344	cg3345	yes^h^
CgLP14^f^	3213531	▷ **⌇** ⋂ ◄	10	cg3365 (*rmpC*)	cg3364 (*trpA*)^g^	–
CgLP15	3229705	◁ **⌇** ⋂ ◄	10	cg3385 (*rhcD2*)	cg3384	–
CgLP16	3248838	◁ **⌇** ⋂ ◄	40	cg3397	cg3396	–

**a.** Referring to the *C. glutamicum* ATCC 13032 complete genome NCBI reference sequence: NC_006958.1.

**b. ⌇** = CgLP; ⋂ = Terminator loop; ◄, ► = upstream gene; ◁, ▷ = downstream gene; arrowheads indicate direction of adjacent genes.

**c.** Spacer between the predicted end of terminator site (Pfeifer‐Sancar *et al*., 2013) and the CgLP position.

**d.** Delivers the terminator site.

**e.** In succession of the CgLP.

**f.** Directly adjacent to the non‐essential gene cluster [outside location CgLP3 (80 bps), CgLP6 (39 bps), CgLP10 (342 bps), CgLP14 (123 bps)].

**g.** Gene outside (up‐ or downstream) the non‐essential gene cluster (Unthan *et al*., 2014); downstream gene is located inside the non‐essential gene cluster.

**h.** Were used in our laboratories and are evidentially feasible (data not shown).

### **d** **‐**Xylose and **l** **‐**arabinose metabolization in CArXy

To enable growth of *C. glutamicum* on d‐xylose and l‐arabinose as abundant components of the organosolv‐derived hemicellulose fraction, we integrated the synthetic operons P_*tuf* _‐*xylAB*‐T_*rrnB*_ and P_*tuf*_‐*araBAD*‐T_*rrnB*_ into CgLP4 and CgLP12 respectively, yielding the strain CArXy (*C. glutamicum* Δ*pqo* Δ*ilvE* Δ*ldhA* Δ*mdh* CgLP4::(P_*tuf*_‐*xylAB*‐T_*rrnB*_) CgLP12::(P_*tuf*_‐*araBAD*‐T_*rrnB*_); cf. Fig. 1). Cloning, isolation and purification of plasmids, PCR fragments or genomic DNA, and procedures for strain construction are given in the Appendix S1, where a detailed list of the applied bacterial strains, plasmids and oligonucleotides (cf. Table S1) is also provided. In brief, the integration of both synthetic operons into the chromosome harnessed a previously published method (Schäfer *et al*., 1994) for plasmid‐based (pK19*mobsacB*) gene disruption and allelic exchange by homologous recombination. We designed homologous flanking regions of > 500 bps to specifically locate the additional genetic information to designated CgLPs. The two synthetic operons express the *xylAB* genes encoding XylA (xylose isomerase) of *Xanthomonas campestris* and XylB (xylulokinase) of *C. glutamicum* and *araBAD* encoding AraB (l‐ribulokinase), AraA (l‐arabinose isomerase) and AraD (l‐ribulose‐5‐phosphate 4‐epimerase) of *E. coli* MG1655 under control of the constitutive promoter of the *C. glutamicum* elongation factor EF‐TU (cg0587, P_*tuf*_) and are terminated by the *E. coli rrnB* operon terminator (T_*rrnB*_) respectively, following already published operon architectures (Schneider *et al*., 2011; Meiswinkel *et al*., 2013).

**Figure 2:**
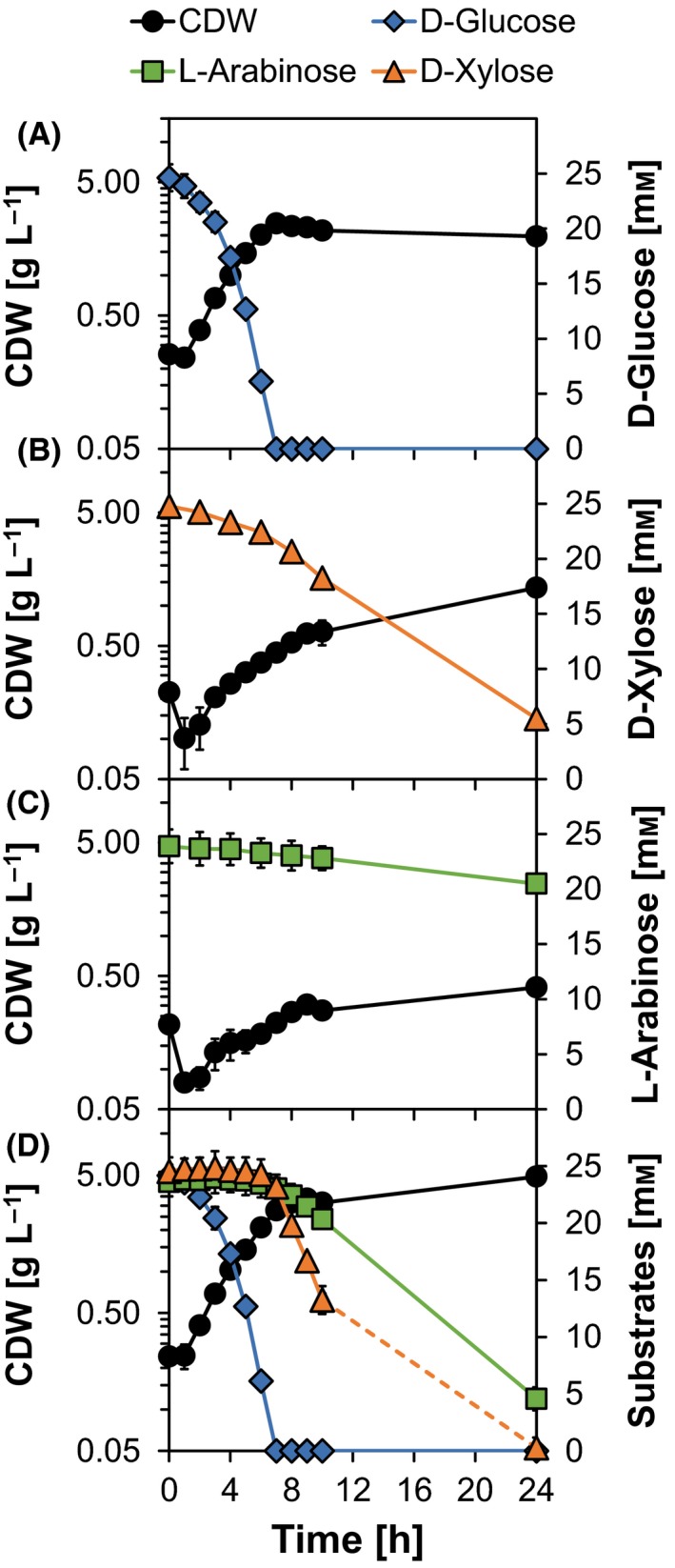
Shaking flask cultivations of the strain CArXy (*C. glutamicum* Δ*pqo* Δ*ilvE* Δ*ldhA* Δ*mdh* CgLP4::(P_*tuf*_ ‐*xylAB*‐T_*rrnB*_) CgLP12::(P_*tuf*_ ‐*araBAD*‐T_*rrnB*_)) in a modified CGXII minimal medium based on the literature (Eikmanns *et al*., 1991; Keilhauer *et al*., 1993) with either combined or single supplementation of 25 mM d‐glucose, d‐xylose and l‐arabinose. Bacterial growth (cell dry weight, CDW) and substrate consumption are depicted over time. Cultivations were performed in 50 ml medium in 500 ml baffled shaking flasks on a rotary shaker at 120 rpm and 30 °C. Detailed information concerning strain construction, medium, seed train and cultivation conditions is given in the Appendix S1. Error bars represent the standard deviation (SD) of three independent experiments.

First, we characterized growth of *C. glutamicum* CArXy in shaking flask cultivations for single and combined metabolization of d‐glucose, d‐xylose and l‐arabinose. CArXy reached a growth rate (μ) of 0.39 ± 0.03 h^−1^, a biomass/substrate yield (Y_X/S_) of 0.52 ± 0.02 g CDW per g d‐glucose and showed a biomass‐specific uptake rate (q_S_) of 4.18 ± 0.16 mmol d‐glucose per g CDW per h (cf. Fig. 2A). All growth parameters were identical to previously described values (Buchholz *et al*., 2014) for the wild type of *C. glutamicum* and indicate that integration of both synthetic operons does not negatively interfere with the strain's vitality under standard cultivation conditions. Furthermore, *C. glutamicum* CArXy grew on d‐xylose and l‐arabinose with rates of 0.18 ± 0.02 h^−1^ and 0.16 ± 0.01 h^−1^, respectively (cf. Fig. 2B, C). Previous studies using plasmid‐based expression of *araBAD* (Schneider *et al*., 2011) or *xylAB* (Meiswinkel *et al*., 2013) yielded maximal rates of 0.31 h^−1^ or 0.20 h^−1^ respectively. In our experiments, a full consumption of the pentoses was not achieved at the end of cultivation (78 ± 7% of d‐xylose and 14 ± 4% of l‐arabinose metabolized). Poor l‐arabinose uptake can be explained by a high Monod constant (Schneider *et al*., 2011) and could be overcome by additional expression of the transporter *araE*, which was shown to also improve d‐xylose consumption (Sasaki *et al*., 2009). Combined supplementation of d‐glucose, d‐xylose and l‐arabinose showed a clear preference for the consumption of the hexose compared to the pentoses (cf. Fig. 2D), a fact that has been described previously for *C. glutamicum* (e.g. Kawaguchi *et al*., 2008; Radek *et al*., 2014). In contrast to the isomerase pathway, the Weimberg pathway enables a more carbon efficient utilization of d‐xylose and allows a parallel consumption of d‐xylose and d‐glucose in *C. glutamicum* (Radek *et al*., 2014, 2016). However, the maximal net generated biomass (4.7 ± 0.4 g CDW L^−1^) was doubled with respect to sole d‐glucose (2.2 ± 0.1 g CDW L^−1^), and the higher cell density allowed a full consumption of d‐xylose and 80% of l‐arabinose within the given cultivation time (cf. Fig. 2D).

In summary, the strain CArXy functionally expresses the synthetic operons in the identified CgLPs enabling d‐xylose and l‐arabinose metabolization without negatively influencing the cell's general viability under given conditions.

### Aerobic growth on the hemicellulose fraction

**Figure 3:**
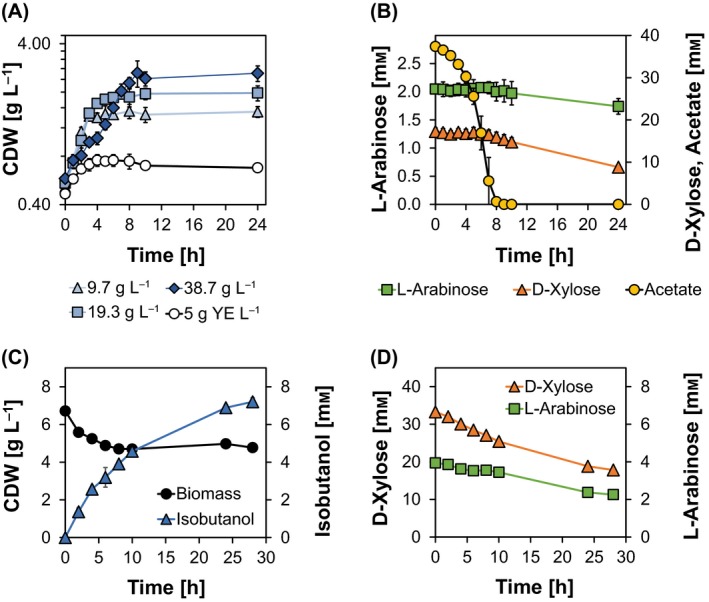
Aerobic cultivation (A, B) of the strain CArXy (*C. glutamicum* Δ*pqo* Δ*ilvE* Δ*ldhA* Δ*mdh* CgLP4::(P_*tuf*_‐*xylAB*‐T_*rrnB*_) CgLP12::(P_*tuf*_‐*araBAD*‐T_*rrnB*_)) and anaerobic isobutanol production (C, D) with CIsArXy (CArXy harbouring pJC4*ilvBNCD*‐*pntAB* and pBB1*kivd*‐*adhA*) using the hemicellulose fraction (HF). A. CArXy was cultivated in CGXII minimal medium supplemented with 5 g YE L^−1^ as reference (open circles) and variable concentrations of hemicellulose fraction (HF) [9.7 g HF L^−1^ (triangles), 19.3 g HF L^−1^ (squares) and 38.7 g HF L^−1^ (diamonds)] + 5 g YE L^−1^. B. Consumption of acetate (circles), d‐xylose (triangles) and l‐arabinose (squares) is depicted for the respective experiment using 38.7 g HF L^−1^. C. Zero‐growth isobutanol production was realized with the strain CIsArXy using 77.3 g HF L^−1^ + 5 g YE L^−1^ in sealed 100 ml flasks containing 50 mL CGXII medium. D. Metabolization of d‐xylose and l‐arabinose during the incubation is shown. Error bars represent SD of three independent experiments. Detailed information concerning strain construction, medium, seed train and cultivation conditions is given in the Appendix S1.

The aqueous hemicellulose fraction (HF) was derived from a beech wood ethanol/water organosolv processing after lignin precipitation (without enzymatic hydrolysis and further purification procedures) as a black liquor with high viscosity (Ludwig *et al*., 2014). A description of the short pretreatment procedure extracting water‐soluble compounds is given in the Appendix S1. To investigate aerobic growth of *C. glutamicum* CArXy (cf. Table S1) on the HF, shaking flask cultivations were performed (cf. Fig. 3A, B, Fig. S2). In contrast to previous studies, in which engineered *C. glutamicum* was shown to proliferate on aci d‐pretreated lignocelluloses such as rice straw and wheat bran in minimal medium (Gopinath *et al*., 2011), growth in the presence of organosolv‐derived HF was only manifested upon additional supplementation of 5 g of yeast extract (YE) L^−1^ (data not shown) and was therefore included in all following experiments. In minimal medium with 5 g YE L^−1^ and 9.7 g HF L^−1^, 19.3 g HF L^−1^ or 38.7 g HF L^−1^ combined with 5 g YE L^−1^, CArXy showed growth rates of 0.14 ± 0.03 h^−1^, 0.34 ± 0.02 h^−1^, 0.33 ± 0.01 h^−1^ and 0.17 ± 0.02 h^−1^ and maximal net generated biomasses of 0.29 ± 0.06, 1.02 ± 0.15, 1.50 ± 0.11 and 2.19 ± 0.41 g CDW L^−1^ respectively. A consecutive consumption of acetate and the pentoses d‐xylose and l‐arabinose was found, and the depletion of acetate coincided with an arrest of the exponential growth phase (cf. Fig. 3A, B, Fig. S2).

Although substrate consumption is still improvable, we show the capability of *C. glutamicum* to grow efficiently on HF which in general opens the opportunity to exploit this biorefinery side stream for microbial production of chemicals and fuels.

### Two‐stage isobutanol production

To prove our concept, we aimed to utilize HF for the production of isobutanol under anaerobic conditions. Therefore, we transformed CArXy with the plasmids pJC4*ilvBNCD*‐*pntAB* and pBB1*kivd*‐*adhA*, which enabled isobutanol production in *C. glutamicum* (cf. Table S1, Blombach *et al*., 2011). Then, the resulting strain CIsArXy was applied in a zero‐growth production processes (Lange *et al*., 2016), where an aerobic stage was implemented to generate biomass that is used in a subsequent anaerobic, growth‐arrested phase to produce isobutanol at high cell densities (cf. Fig. 3C, D). Under anaerobic conditions, we observed a simultaneous metabolization of d‐xylose and l‐arabinose (cf. Fig. 3D, acetate was not consumed cf. Fig. S3), which directly served as substrate for isobutanol production (cf. Fig. 3C). No significant production of lactate or succinate (< 0.4 mM) was found. About 15.5 ± 0.6 mM (46 ± 1%) and 1.7 ± 0.0 mM (43 ± 1%) of d‐xylose and l‐arabinose were metabolized respectively, and CIsArXy produced 7.2 ± 0.2 mM of isobutanol within 28 h of cultivation. With respect to the analysed pentoses, a carbon molar product/substrate yield (Y_P/S_) of 0.31 ± 0.02 C‐mol isobutanol per C‐mol substrate (d‐xylose + l‐arabinose) was achieved, which is already in the range of d‐glucose‐based processes with engineered *C. glutamicum* strains (0.15–0.52 C‐mol C‐mol^−1^; Blombach *et al*., 2011; Smith *et al*., 2010; Yamamoto *et al*., 2013). Isobutanol production based on the pentoses d‐xylose and l‐arabinose has so far not been demonstrated and therefore represents a promising example for the valorization of HF within a novel value chain. As a future perspective, a dual‐phase process (Lange *et al*., 2016) is apparent, where an aerobic growth based on acetate within the HF would be directly followed by an anaerobic isobutanol production phase based on the remaining pentoses.

## Conclusions

In the presented study, we systematically identified 16 landing pads, which represent prominent loci for chromosomal integration of additional genetic information in *C. glutamicum*. As a proof of concept, we integrated synthetic operons into two CgLPs that enabled growth on d‐xylose and l‐arabinose as well as on a so far unexploited hemicellulose fraction derived from beech wood organosolv processing. For the first time, we showed isobutanol production with engineered *C. glutamicum* based on pentoses within this fraction. The work demonstrates the suitability to microbially convert complex side streams to valuable products, enabling a holistic exploitation of renewable resources in biorefinery approaches. Moreover, the proposed chromosomal integration loci can be prospectively used as basis for metabolic engineering in future studies.

## Conflict of interest

None declared.

## Supporting information


**Appendix S1.** Material and Methods.
**Fig. S1.** Novel proposed *C. glutamicum* landing pads (red, CgLPs) located in the genome of *C. glutamicum* ATCC 13032 (NCBI reference sequence NC_006958.1).
**Fig. S2.** Aerobic cultivation of the strain CArXy (*C. glutamicum* Δ*pqo* Δ*ilvE* Δ*ldhA* Δ*mdh* CgLP4::(P_*tuf*_ ‐*xylAB*‐T_*rrnB*_) CgLP12::(P_*tuf*_ ‐*araBAD*‐T_*rrnB*_)) in CGXII minimal medium supplemented with 5 g yeast extract (YE) L^−1^ as reference (open circles) and variable concentrations of hemicellulose fraction (HF, circles) [9.7 g HF L^−1^ (A), 19.3 g HF L^−1^ (B) and 38.7 g HF L^−1^ (C)] + 5 g YE L^−1^.
**Fig. S3.** Course of acetate concentration during the anaerobic isobutanol production with the strain CIsArXy (CArXy harboring pJC4*ilvBNCD*‐*pntAB* and pBB1*kivd*‐*adhA*) using the HF (cf. Fig. 3). Error bars represent SD of three independent experiments.
**Table S1.** List of bacterial strains, plasmids and oligonucleotides.
