# Supplementary material for: Harnessing novel chromosomal integration loci to utilize an organosolv‐derived hemicellulose fraction for isobutanol production with engineered Corynebacterium glutamicum
Source: Microb Biotechnol. 2017 Nov 8;11(1):257–63. doi: 10.1111/1751-7915.12879 (PMC5743825; doi:10.1111/1751-7915.12879)
Supplement: Supplementary file 1 — Appendix S1. Material and Methods. Fig. S1. Novel proposed C. glutamicum landing pads (red, CgLPs) located in the genome of C. glutamicum ATCC 13032 (NCBI reference sequence NC_006958.1). Fig. S2. Aerobic cultivation of the strain CArXy (C. glutamicum Δpqo ΔilvE ΔldhA Δmdh CgLP4::(Ptuf ‐xylAB‐TrrnB) CgLP12::(Ptuf ‐araBAD‐TrrnB)) in CGXII minimal medium supplemented with 5 g yeast extract (YE) L−1 as reference (open circles) and variable concentrations of hemicellulose fraction (HF, circles) [9.7 g HF L−1 (A), 19.3 g HF L−1 (B) and 38.7 g HF L−1 (C)] + 5 g YE L−1. Fig. S3. Course of acetate concentration during the anaerobic isobutanol production with the strain CIsArXy (CArXy harboring pJC4ilvBNCD‐pntAB and pBB1kivd‐adhA) using the HF (cf. Fig. 3). Error bars represent SD of three independent experiments. Table S1. List of bacterial strains, plasmids and oligonucleotides. [file MBT2-11-257-s001.docx]

**Supplementary Material**

The following information is provided to the article

**Harnessing novel chromosomal integration loci to utilize an organosolv-derived hemicellulose fraction for isobutanol production with engineered *Corynebacterium glutamicum***

in *Microbial Biotechnology*

Julian Lange^a^

Felix Müller^a^

Ralf Takors^a^

Bastian Blombach^a^^[[1]](#footnote-1)^

**Running Title:**

Novel *C. glutamicum* LPs for hemicellulose utilization

# Supplementary Material and Methods

## Plasmids, PCR fragments and genomic DNA

General molecular biology methods (e.g. PCR, restriction, agarose gel electrophoresis) were conducted as described in literature (Sambrook and Russell, 2001). Isolation and purification of plasmids, PCR fragments or genomic DNA was accomplished after the manufacturer’s instructions of E.Z.N.A.® Plasmid Mini Kit I (Omega Bio-tek, Inc., Norcross, USA), NucleoSpin® Gel and PCR Clean-up (Macherey-Nagel GmbH & Co. KG, Düren, Germany) and DNeasy Blood & Tissue Kit (QIAGEN, Hilden, Germany).

## Plasmid Cloning Procedure

For cloning of plasmids the necessary fragments were amplified via PCR (Phusion Hot Start II HF DNA Polymerase, Thermo Fisher Scientific Inc., Waltham, USA; Biometra TAdvanced thermocycler, Analytik Jena, Jena, Germany) with the use of designed oligonucleotides (biomers.net GmbH, Ulm, Germany).

A full list of the applied bacterial strains, plasmids and oligonucleotides is provided in Tab. S1. To release *C. glutamicum* Δ*aceE* Δ*pqo* Δ*ilvE* Δ*ldhA* Δ*mdh* (Blombach *et al.*, 2011) of its auxotrophy for acetate, we restored the *aceE* gene encoding the E1 subunit of the pyruvate dehydrogenase complex with the plasmid pJUL*aceE* (8918 bps). The plasmid was constructed by ligation of SalI restricted pK19*mobsacB* (Schäfer *et al.*, 1994) and the amplified *aceE* fragment (template: *C. glutamicum* wild type chromosomal DNA, primer pair: aceE1/aceE2) using the T4 DNA Ligase (Thermo Fisher Scientific Inc., Waltham, USA). Alkaline phosphatase treatment was conducted standardly after restriction (Thermo Fisher Scientific Inc., Waltham, USA). The cloned fragment was sequenced with the primers pK19seqfw, aceEseq1, aceEseq2 and pK19seqrv (GATC Biotech AG, Konstanz, Germany).

The plasmids pJUL*xylAB* and pJUL*araBAD* were also constructed based on pK19*mobsacB*. Oligonucleotides provided an overlap of the adjacent fragments during amplification. To integrate the synthetic *xylAB* and *araBAD* operons into the chromosome of *C. glutamicum*, upstream (Flank1) and downstream flanks (Flank2) for homologous recombination were of at least 500 bps length and designed to locate into the newly identified landing pads CgLP4 and CgLP12 (cf. Tab. 1), respectively. To clone pJUL*xylAB*, the backbone pK19*mobsacB* was linearized by single restriction with NheI. Pieces of the integrative construct Flank1-P*_tuf_*-*xylAB*-T*_rrnB_*-Flank2 were amplified from *C. glutamicum* chromosomal DNA with the primers P1/P2 (P*_tuf_*), xyl1/xyl2 (Flank1) and xyl5/xyl6 (Flank2) and using pEKEx3-*xylA_Xc_*-*xylB_Cg_* (Meiswinkel *et al.*, 2013) as template with the primer pair xyl3/xyl4 (*xylAB*-T*_rrnB_*). To construct pJUL*araBAD*, pK19*mobsacB* was linearized with PaeI/NheI restriction enzymes. Fragments for the desired construct Flank1-P*_tuf_*-*araBAD*-T*_rrnB_*-Flank2 were amplified via PCR using *C. glutamicum* chromosomal DNA as template with the primer pairs P1/P2 (P*_tuf_*, promoter of the elongation factor EF-TU [cg0587]), ara1/ara2 (Flank1) and ara5/ara6 (Flank2) and pVWEx1-*araBAD* (Schneider *et al.*, 2011) as template with the primer pairs ara3/ara4 (*araBAD*-T*_rrnB_*) that include the strong terminator of the *E. coli rrnB* operon (Brosius *et al.*, 1981). Cloning of fragments and backbone was accomplished after Gibson et al. (2009) via isothermal assembly. As the fragment length varied strongly, SOEing PCR was used to reduce the number of applied fragments and approximate fragment sizes (Horton *et al.*, 1989). The isothermal assembly mix was directly conferred to *E. coli* DH5α by electroporation. The plasmid’s integrity was verified by sequencing with primers pK19seqfw and pK19seqrv and xylseq1-xylseq6 for pJUL*xylAB* (10237 bps) and araseq1-araseq8 for pJUL*araBAD* (11230 bps).

## Transformation of Strains with Plasmids via Electroporation

Electrocompetent cells were produced by following established protocols for *E. coli* (Dower *et al.*, 1988) and *C. glutamicum* (Tauch *et al.*, 2002). Purified plasmids were then introduced into *E. coli* and *C. glutamicum* via electroporation (Dower *et al.*, 1988; Liebl *et al.*, 1989; van der Rest *et al.*, 1999). Plasmid harboring strains were afterwards selected on 2x yeast tryptone (2x YT, Sambrook and Russell, 2001) or BHI (Bacto^TM^ brain heart infusion, Becton, Dickinson and Company, New Jersey, USA) supplemented with 91 g sorbitol L^‑1^ agar plates (Eggeling and Reyes, 2005) including antibiotics as indicated in Tab. S1 for *E. coli* or *C. glutamicum*, respectively.

## Strain Construction

To construct the *aceE* restored strain *C. glutamicum* Δ*pqo* Δ*ilvE* Δ*ldhA* Δ*mdh* and the pentose utilizing strain CArXy (*C. glutamicum* Δ*pqo* Δ*ilvE* Δ*ldhA* Δ*mdh* CgLP4::(P*_tuf_*-*xylAB*-T*_rrnB_*) CgLP12::(P*_tuf_*-*araBAD*-T*_rrnB_*)), markerless gene integration via pJUL*aceE* or pJUL*xylAB* and pJUL*araBAD* was conducted consecutively after Schäfer et al. (1994). The strains were verified via colony PCR (primer pairs: aceE3/aceE4 and ara1/P2, xyl1/P2, xylseq4/xyl6 and xylseq3/xylseq5, araseq3/P1, araseq6/ara6).

For isobutanol production pJC4*ilvBNCD*-*pntAB* and pBB1*kivd*-*adhA* (Blombach *et al.*, 2011) were introduced into CArXy yielding the strain CIsArXy that was confirmed to contain the plasmids by colony PCR (primer pairs: iso1/iso2 and iso3/iso4).

## Cultivation Conditions

*E. coli* and *C. glutamicum* were cultivated in 2x yeast tryptone (2x YT complex medium (Sambrook and Russell, 2001). For growth on semi-solid media 18 g agar L^-1^ was added. Incubation was conducted on rotary shakers at 120 rpm and 37 °C or 30 °C for *E. coli* or *C. glutamicum*, respectively. Minimal medium cultivations with *C. glutamicum* were performed in a modified CGXII medium at pH 7.4 based on literature (Eikmanns *et al.*, 1991; Keilhauer *et al.*, 1993). The medium comprised per liter: 5 g (NH_4_)_2_SO_4_, 5 g urea, 21 g 3-(N-morpholino) propane sulphonic acid (MOPS), 1 g KH_2_PO_4_, 1 g K_2_HPO_4_, 0.25 g MgSO_4_ · 7 H_2_O, 10 mg CaCl_2_, 10 mg MnSO_4_ · H_2_O, 16.4 mg FeSO_4_ · 7 H_2_O, 1 mg ZnSO_4_ · 7 H_2_O, 0.2 mg CuSO_4_ · 5 H_2_O, 0.02 mg NiCl_2_ · 6 H_2_O, and 0.2 mg biotin.

In cultivations using the hemicellulose fraction (HF) 5 g yeast extract L^-1^ (BBL™ Yeast Extract, BD, New Jersey, USA) was added as further supplement. For all *C. glutamicum* Δ*ilvE* derivatives 2 mM valine, 2 mM leucine and 2 mM isoleucine were supplemented in any instance (Merck Millipore, Billerica, Massachusetts, USA). For plasmid selection, antibiotics were added according to Tab. S1: 100 µg spectinomycin (Spec) mL^-1^, 50 µg kanamycin (Kan) mL^-1^ or 6 µg chloramphenicol (Cm) mL^-1^.

Aerobic cultivations of *C. glutamicum* were conducted as follows: 2x YT agar plates (2‑3 days), 5 mL 2x YT in a test tube (6-8 h), 50 mL 2x TY in a 500 mL baffled shaking flask (12-15 h). From this culture a desired amount of cells was harvested by centrifugation and resuspended in 0.9 % (w/v) NaCl to inoculate a 50 mL CGXII culture in 500 mL baffled shaking flasks to approximately 0.25 g CDW L^-1^ in defined medium or 0.5 g CDW L^-1^ where yeast extract and hemicellulose fraction were supplemented.

The two-stage isobutanol production (Lange *et al.*, 2016) involving the HF was conducted with the following seed train: 2-3 days on 2x YT agar plate, 14-16 h in 5 mL 2x YT, 6‑8 h in 50 mL 2x YT, and 14‑16 h in 50 mL CGXII + 40 g D‑glucose L^-1^ (starting biomass 0.25 g CDW L^-1^). For anaerobic isobutanol production the desired amount of cells was harvested as described above and inoculated through a syringe into sealed 100 mL shaking flask without baffles containing 50 mL CGXII medium to a starting cell density of 6.7 g CDW L^-1^. Samples for analytics were taken aseptically via a syringe to prevent a gas exchange.

## Hemicellulose Fraction and Pretreatment Procedures

For cultivation purposes, the desired amount of hemicellulose fraction (HF) was weighed and mixed with 5 mL pure water. The suspension was clarified in a centrifugal step at 4500 rcf for 30 min (Centrifuge 5804 R, Rotor: A-4-44, Eppendorf AG, Hamburg, Germany) and used for cultivation. In anaerobic shaking flasks, the clarified HF substrate was added to the CGXII medium and the pH adjusted to 7.4. The entire medium was then sterilized via membrane filters (Rotilabo®-syringe filters, CME Filter 0.45 µm, Carl Roth GmbH + Co. KG, Karlsruhe, Germany) and final supplements added aseptically.

## Analytical Methods

Bacterial growth was followed by optical density (OD) analysis of a biosuspension aliquot at 600 nm (Ultrospec 10 Cell Density Meter, GE Healthcare Europe GmbH, Freiburg, Germany). A correlation coefficient α of 0.22 g L^-1^ to the cell dry weight (CDW) concentration was determined in reference cultivations (CDW = α · OD_600_).

Analysis of metabolites (D-glucose, D-xylose, L-arabinose, acetate, isobutanol) was conducted via HPLC as previously described (Buchholz et al., 2013) using an external 8‑level standard calibration of each analyte for quantification. For isobutanol analysis the run time was extended from published 45 min (Buchholz et al., 2013) to 52 min.

## Calculations

Calculations of the growth rate (µ) in the exponential phase were performed by linear regression in semi-logarithmic plots of the OD_600_ above the cultivation time. Molar biomass/substrate yields (Y_X/S_) and C-molar product/substrate yields (Y_P/S_) were calculated via linear regression in a biomass versus substrate or product versus substrate concentration diagram, respectively. The biomass specific uptake rate (q_S_) was determined by the correlation q_S_ = µ / Y_X/S_ for the exponential growth phase. For all graphs and calculations errors are given by the standard deviation (SD) of at least three independent experiments.

# Supplementary Figures

## Fig. S1

*_
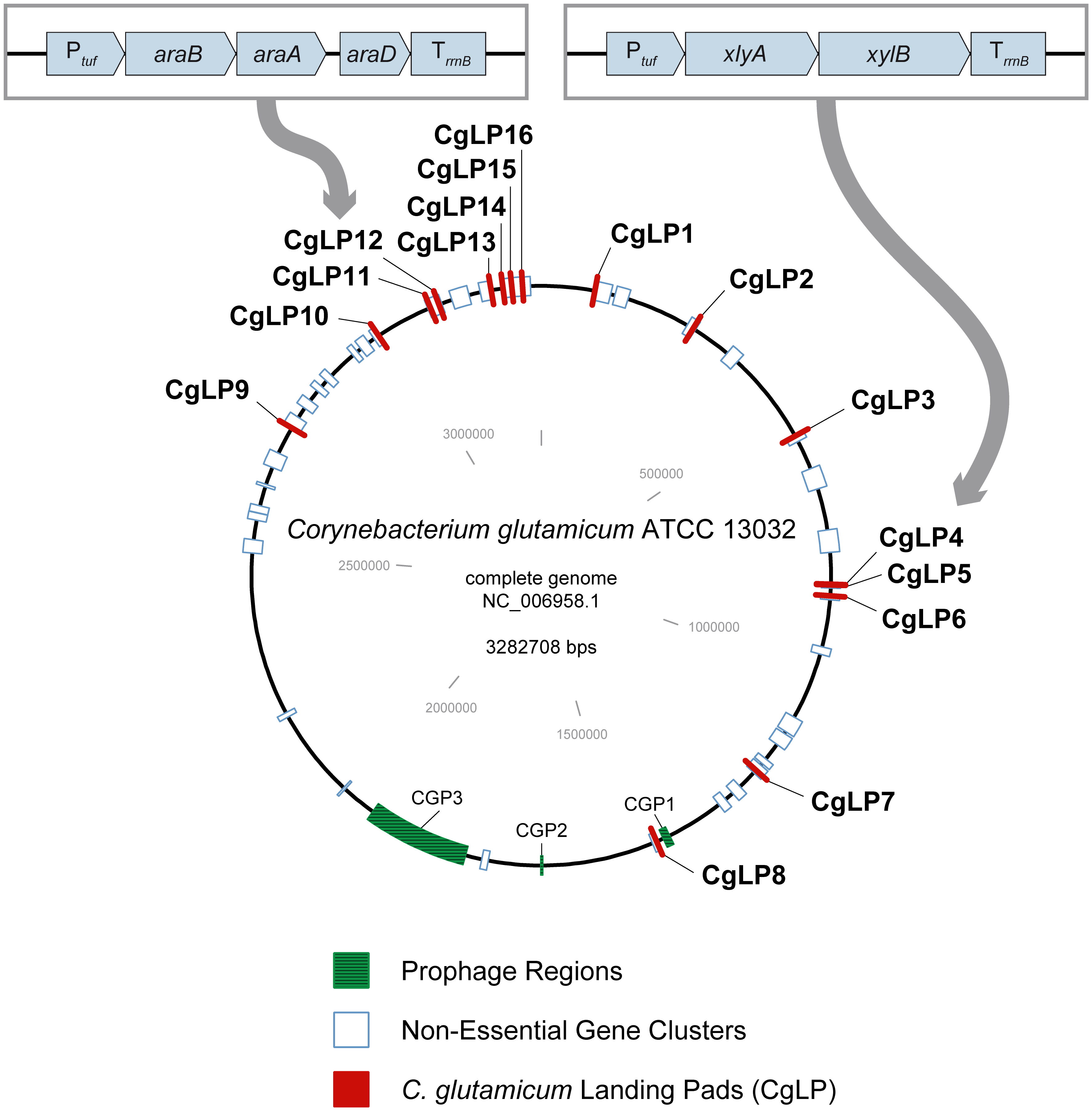
_*

Fig. S1. Novel proposed *C. glutamicum* landing pads (red, CgLPs) located in the genome of *C. glutamicum* ATCC 13032 (NCBI reference sequence NC_006958.1). Exact position of each CgLP is given in Tab. 1. Additionally, prophage regions of *C. glutamicum* [CGP1 (cg1507-cg1524), CGP2 (cg1746-cg1752), and CGP3 (cg1890-cg2071)] (shaded green, Kalinowski, 2005) and non-essential gene clusters (open box, Unthan *et al.*, 2014) are shown. For D-xylose and L-arabinose valorization the synthetic operons P*_tuf_*-*xylAB*-T*_rrnB_* and P*_tuf_*‑*araBAD*‑T*_rrnB_* were integrated into the CgLP4 and CgLP12 as indicated, respectively.

## Fig. S2


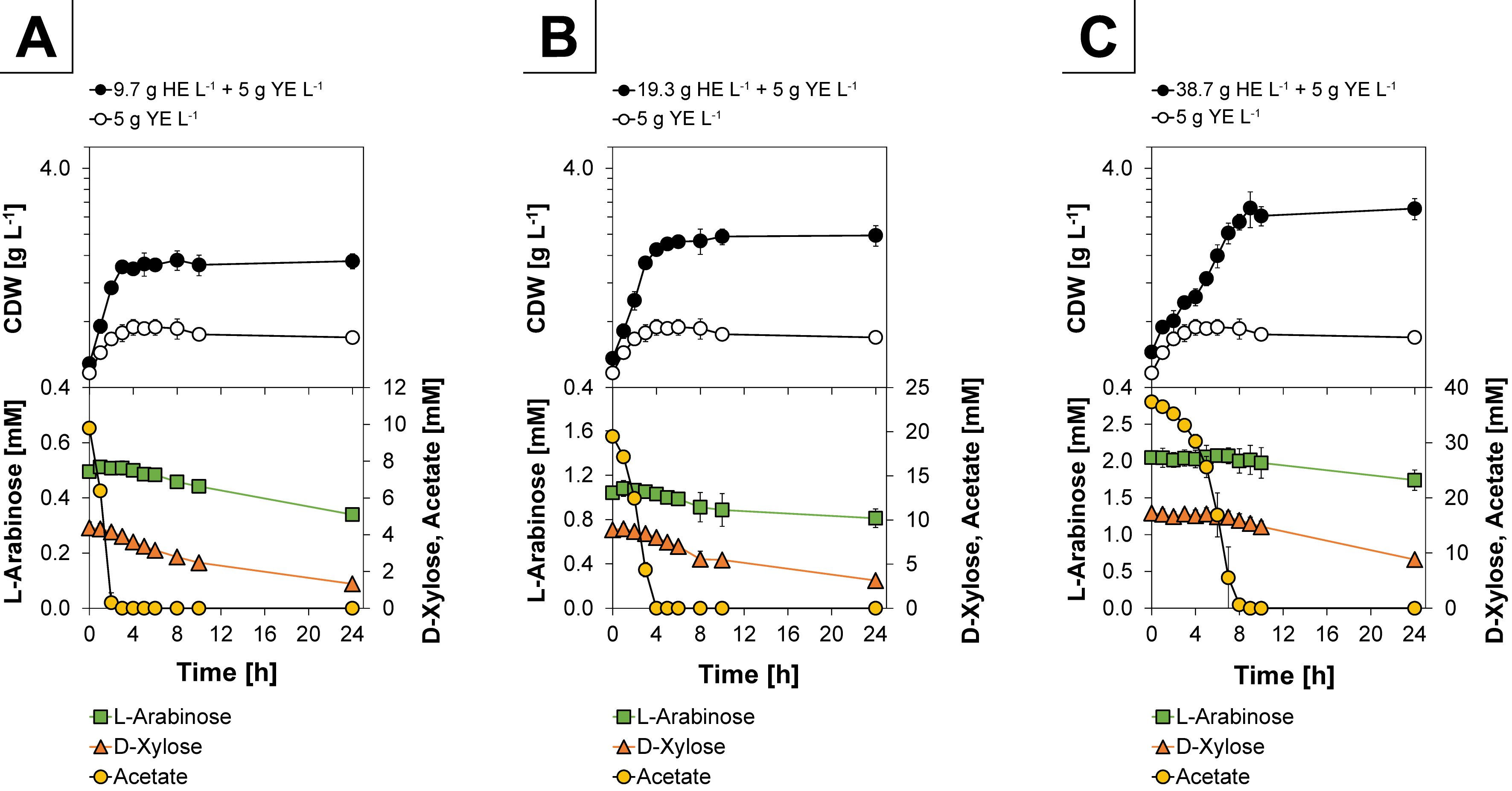


Fig. S2. Aerobic cultivation of the strain CArXy (*C. glutamicum* Δ*pqo* Δ*ilvE* Δ*ldhA* Δ*mdh* CgLP4::(P*_tuf_*-*xylAB*-T*_rrnB_*) CgLP12::(P*_tuf_*-*araBAD*-T*_rrnB_*)) in CGXII minimal medium supplemented with 5 g yeast extract (YE) L^-1^ as reference (open circles) and variable concentrations of hemicellulose fraction (HF, circles) [9.7 g HF L^-1^ (A), 19.3 g HF L^-1^ (B) and 38.7 g HF L^-1^ (C)] + 5 g YE L^-1^. Consumption of acetate (circles), D-xylose (triangles) and L‑arabinose (squares) is depicted for the respective experiment. Error bars represent SD of three independent experiments.

## Fig. S3


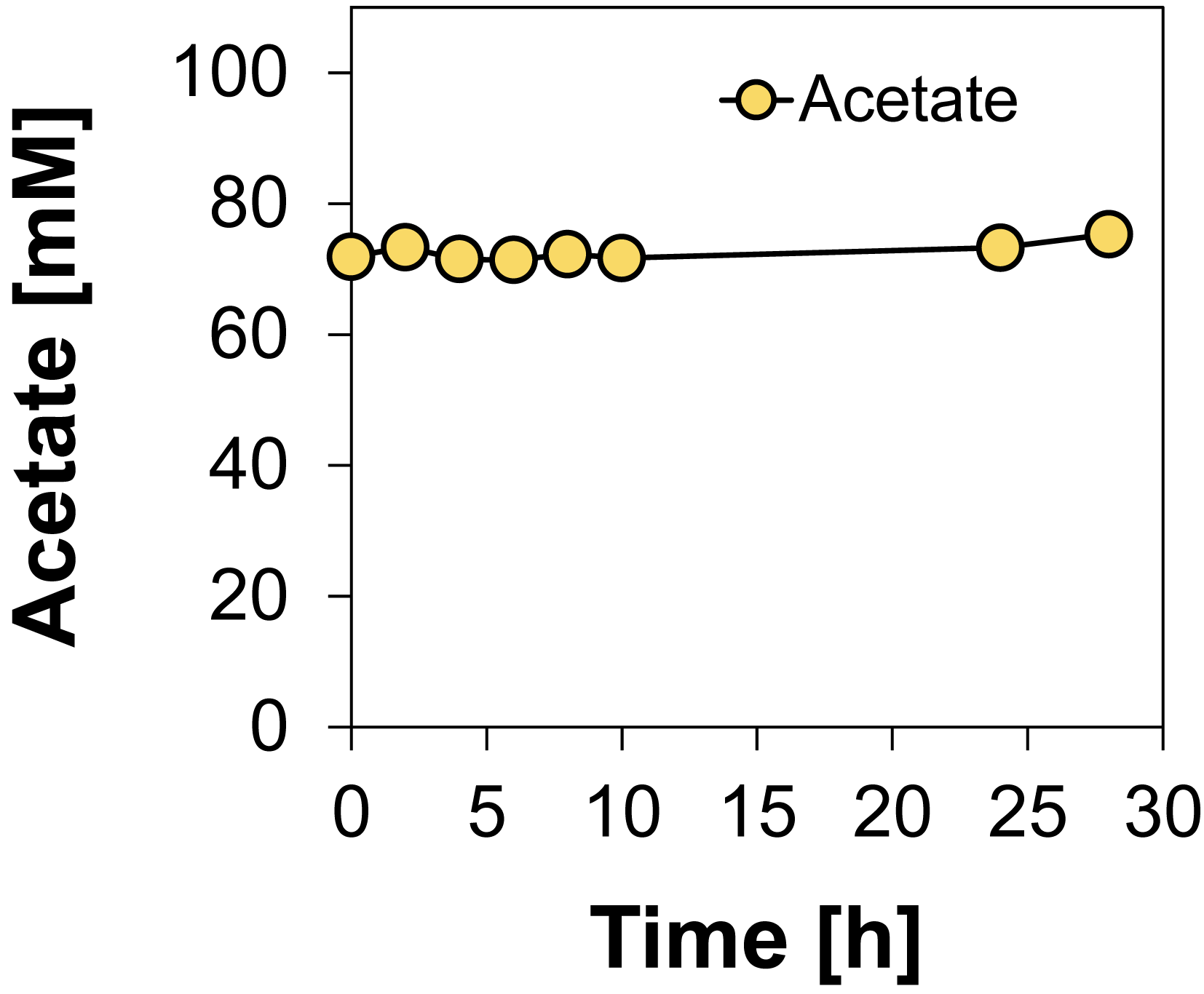


Fig. S3. Course of acetate concentration during the anaerobic isobutanol production with the strain CIsArXy (CArXy harboring pJC4*ilvBNCD*-*pntAB* and pBB1*kivd*-*adhA*) using the HF (cf. Fig. 3). Error bars represent SD of three independent experiments.

## Tab. S1

Tab. S1. List of bacterial strains, plasmids and oligonucleotides. Concerning oligonucleotides the homology region for Gibson assembly (underlined) and restrictions sites (bold) are indicated and described in parenthesis.

| **Strain, plasmid, or**  **oligonucleotide** | **Relevant characteristics, purpose, genotype or sequence** | **Source or reference** |
| --- | --- | --- |
| **Strains** |  |  |
| *Escherichia coli* DH5α | F^-^ Φ80*lacZ*ΔM15 Δ(*lacZYA-argF*) U169 *endA1 recA1 hsdR17* (rk^-^, mk^+^) *supE44 thi-1 gyrA96 relA1 phoA* | (Hanahan, 1983) |
| *Corynebacterium glutamicum* ATCC13032 | Wild type | American Type Culture Collection |
| *C. glutamicum* | Δ*aceE* Δ*pqo* Δ*ilvE* Δ*ldhA* Δ*mdh* | (Blombach *et al.*, 2011) |
| *C. glutamicum* | Δ*pqo* Δ*ilvE* Δ*ldhA* Δ*mdh* | This study |
| CArXy | *C. glutamicum* Δ*pqo* Δ*ilvE* Δ*ldhA* Δ*mdh* CgLP4::(P*_tuf_*‑*xylAB*-T*_rrnB_*) CgLP12::(P*_tuf_*‑*araBAD*-T*_rrnB_*) | This study |
| CIsArXy | CArXy harboring the plasmids pJC4*ilvBNCD*-*pntAB* and pBB1*kivd*-*adhA*, Kan^R^, Cm^R^ | This study |
|  |  |  |
| **Plasmids** |  |  |
| pK19*mobsacB* | For gene integration (*lacZ*α, RP4 *mob*, *oriV_E. coli_*, *sacB_Bacillus subtilis_*, Kan^R^) | (Schäfer *et al.*, 1994) |
| pEKEx3-*xylA_Xc_*-*xylB_Cg_* | Vector for expression of the genes encoding XylA (xylose isomerase) of *Xanthomonas campestris* and XylB (xylulokinase) of *C. glutamicum*, Spec^R^ | (Meiswinkel *et al.*, 2013) |
| pVWEx1-*araBAD* | Vector for expression of the genes encoding AraB (L‑ribulokinase), AraA (L-arabinose isomerase) and AraD (L‑ribulose-5‑phosphate 4-epimerase) of *E. coli* MG1655, Kan^R^ | (Schneider *et al.*, 2011) |
| pBB1*kivd*-*adhA* | Constitutive expression of the genes encoding Kivd (2‑ketoacid decarboxylase) of *L. lactis* and AdhA (alcohol dehydro-genase) of *C. glutamicum*, Cm^R^ | (Blombach *et al.*, 2011) |
| pJC4*ilvBNCD-pntAB* | Constitutive expression of the genes encoding IlvBN (acetohydroxyacid synthase), IlvC (acetohydroxyacid isomeroreductase), IlvD (dihydroxyacid dehydratase) of *C. glutamicum* and PntAB (membrane bound transhydrogenase) of *E. coli*, Kan^R^ | (Blombach *et al.*, 2011) |
| pJUL*aceE* | Restoration of the *aceE* gene in *C. glutamicum* Δ*aceE* Δ*pqo* Δ*ilvE* Δ*ldhA* Δ*mdh*, pK19*mobsacB*::*aceE* | This study |
| pJUL*xylAB* | For integration of *xylAB* into CgLP4, pK19*mobsacB*::(Flank1-P*_tuf_*-*xylAB*-T*_rrnB_*-Flank2) | This study |
| pJUL*araBAD* | For integration of *araBAD* into CgLP12, pK19*mobsacB*::(Flank1-P*_tuf_*-*araBAD*-T*_rrnB_*-Flank2) | This study |
|  |  |  |
| **Oligonucleotides** | **5’ 🡪 3’** |  |
| aceE1 | ACGC**GTCGAC**CACCAAAAGGACATCAGACC | Fw primer for pJUL*aceE* (**SalI**) (= delaceE1 (Schreiner *et al.*, 2005) |
| aceE2 | TGCG**GTCGAC**GCGGGATTTATCTGTCCC | Rv primer for pJUL*aceE* (**SalI**) |
| aceE3 | CGGAGGAGACCAACGAGTGGATGGATTCAC | Fw primer to verify *aceE* restoration |
| aceE4 | GTGGGTCACGTCCGAAGAAGTGCTCAC | Rv primer to verify *aceE* restoration |
| aceEseq1 | CCGTGGCATCAAGGACACC | Sequencing primer pJUL*aceE* |
| aceEseq2 | GGCTACCTGCCAGAGCGTCGTG | Sequencing primer pJUL*aceE* |
| P1 | CCACAGGGTAGCTGGTAGTTTG | Fw primer P*_tuf_* for pJUL*xylAB* and pJUL*araBAD* |
| P2 | CATGGTATGTCCTCCTGGACTTC | Rv primer P*_tuf_* for pJUL*xylAB* and pJUL*araBAD* |
| ara1 | GATTACGCCAAGCTT**GCATGC**GAACGTTGAAGACTCCGTCAAAC | Fw primer for Flank1 in pJUL*araBAD* (pK19mobsacB, **PaeI**) |
| ara2 | CTACCAGCTACCCTGTGGAATATGCCGATTGCAAGAAACGAGAAG | Rv primer for Flank1 in pJUL*araBAD* (P*_tuf_*) |
| ara3 | GTCCAGGAGGACATACCATGGCGATTGCAATTGGCCTC | Fw primer *araBAD* (P*_tuf_*) |
| ara4 | GTGCTGATTTCAACATTTTTTGACTGGTCCTACTCAGGAGAGCGTTC | Rv primer *araBAD* (Flank2) |
| ara5 | GAACGCTCTCCTGAGTAGGACCAGTCAAAAAATGTTGAAATCAGCAC | Fw primer for Flank2 in pJUL*araBAD* (*araBAD*) |
| ara6 | GCGGCAGCGTGAA**GCTAGC**GCGCTTCTTTGAAGAGTCTC | Rv primer for Flank2 in pJUL*araBAD* (pK19mobsacB, **NheI**) |
| xyl1 | GAATGGCGCGATAA**GCTAGC**CCGTTCGGCTGACTCCTTC | Fw primer for Flank1 in pJUL*xylAB* (pK19mobsacB, **NheI**) |
| xyl2 | CAAACTACCAGCTACCCTGTGGCATCAAAAAATCCGCCGTTCCTTG | Rv primer for Flank1 in pJUL*xylAB* (P*_tuf_*) |
| xyl3 | GAAGTCCAGGAGGACATACCATGAGCAACACCGTTTTCATC | Fw primer *xylAB* (P*_tuf_*) |
| xyl4 | CGCATCCAAACTCACTTAGTCAATATTATTGAAGCATTTATCAGGG | Rv primer *xylAB* (Flank2) |
| xyl5 | CCTGATAAATGCTTCAATAATATTGACTAAGTGAGTTTGGATGCGGAAG | Fw primer for Flank2 in pJUL*xylAB* (*xylAB*) |
| xyl6 | GCGGCAGCGTGAA**GCTAGC**CTCACTAGTACGCGGATAAATG | Rv primer for Flank2 in pJUL*xylAB* (pK19mobsacB, **NheI**) |
| araseq1 | GGTTCCAGTTTCTGACGC | Sequencing primer pJUL*araBAD* |
| araseq2 | GACTCGCCAGGACAGC | Sequencing primer pJUL*araBAD* |
| araseq3 | CCGGCGCTGCAACGTC | Sequencing primer pJUL*araBAD* |
| araseq4 | GCGATAAAGTTGCCGCAC | Sequencing primer pJUL*araBAD* |
| araseq5 | CGATATGCGCCAATTCGC | Sequencing primer pJUL*araBAD* |
| araseq6 | CGGCGAATATGAGTGGG | Sequencing primer pJUL*araBAD* |
| araseq7 | CTGCGGCGCTAACTGAC | Sequencing primer pJUL*araBAD* |
| araseq8 | CCGTGAAGTCACTCATG | Sequencing primer pJUL*araBAD* |
| xylseq1 | GATTCGCAGGATCTTCCC | Sequencing primer pJUL*xylAB* |
| xylseq2 | CGGACTTCAACGTCGTGG | Sequencing primer pJUL*xylAB* |
| xylseq3 | CGGACTTTGCCAACGGC | Sequencing primer pJUL*xylAB* |
| xylseq4 | CACTGAGCTGAGCACGC | Sequencing primer pJUL*xylAB* |
| xylseq5 | GCCTGACGAGCTGCAC | Sequencing primer pJUL*xylAB* |
| xylseq6 | CCAAGCGAACCGTCCTC | Sequencing primer pJUL*xylAB* |
| pK19seqfw | TAATGCAGCTGGCACGAC | Fw Sequencing primer pJUL*aceE*, pJUL*xylAB* and pJUL*araBAD* (Shah *et al.*, 2016) |
| pK19seqrv | TAATGGTAGCTGACATTCATCCG | Rv Sequencing primer pJUL*aceE*, pJUL*xylAB* and pJUL*araBAD* |
| iso1 | AACTGCAGAACCAATGCATTGGAGGAGACACAACATGTATACAGTAGGAGATTACCTAT | Fw primer verifies pBB1*kivd*-*adhA* (identical to kivdfow (Blombach *et al.*, 2011)) |
| iso2 | CCAATGCATTGGTTCTGCAGTTTTATGATTTATTTTGTTCAGCAAAT | Rv primer verifies pBB1*kivd*-*adhA* (identical to kivd2rev (Blombach *et al.*, 2011)) |
| iso3 | CACTCCCGTTCTGGATAATG | Fw primer verifies pJC4*ilvBNCD-pntAB* (identical to Ptaccheck (Blombach *et al.*, 2011)) |
| iso4 | CCCAAATTCATGTGCCGCTTC | Rv primer verifies pJC4*ilvBNCD-pntAB* |

# References

Blombach, B., Riester, T., Wieschalka, S., Ziert, C., Youn, J.-W., Wendisch, V.F., and Eikmanns, B.J. (2011) *Corynebacterium glutamicum* tailored for efficient isobutanol production. *Appl. Environ. Microbiol.* **77**: 3300–10.

Brosius, J., Dull, T.J., Sleeter, D.D., and Noller, H.F. (1981) Gene organization and primary structure of a ribosomal RNA operon from *Escherichia coli*. *J. Mol. Biol.* **148**: 107–27.

Buchholz, J., Schwentner, A., Brunnenkan, B., Gabris, C., Grimm, S., Gerstmeir, R., et al. (2013) Platform engineering of *Corynebacterium glutamicum* with reduced pyruvate dehydrogenase complex activity for improved production of L-lysine, L‑valine, and 2-ketoisovalerate. *Appl. Environ. Microbiol.* **79**: 5566–75.

Dower, W.J., Miller, J.F., and Ragsdale, C.W. (1988) High efficiency transformation of *E. coli* by high voltage electroporation. *Nucleic Acids Res.* **16**: 6127–45.

Eggeling, L. and Reyes, O. (2005) Experiments. In, Eggeling, L. and Bott, M. (eds), *Handbook of Corynebacterium glutamicum*. CRC Press, Boca Raton, FL, USA, pp. 421–22.

Eikmanns, B.J., Metzger, M., Reinscheid, D., Kircher, M., and Sahm, H. (1991) Amplification of three threonine biosynthesis genes in *Corynebacterium glutamicum* and its influence on carbon flux in different strains. *Appl. Microbiol. Biotechnol.* **34**: 617–22.

Hanahan, D. (1983) Studies on transformation of *Escherichia coli* with plasmids. *J. Mol. Biol.* **166**: 557–80.

Horton, R.M., Hunt, H.D., Ho, S.N., Pullen, J.K., and Pease, L.R. (1989) Engineering hybrid genes without the use of restriction enzymes: gene splicing by overlap extension. *Gene* **77**: 61–8.

Kalinowski, J. (2005) The genomes of amino acid-producing *Corynebacteria*. In, Eggeling, L. and Bott, M. (eds), *Handbook of Corynebacterium glutamicum*. CRC Press, Boca Raton, FL, USA, pp. 37–56.

Keilhauer, C., Eggeling, L., and Sahm, H. (1993) Isoleucine synthesis in *Corynebacterium glutamicum*: molecular analysis of the *ilvB*-*ilvN*-*ilvC* operon. *J. Bacteriol.* **175**: 5595–603.

Lange, J., Takors, R., and Blombach, B. (2016) Zero-growth bioprocesses – A challenge for microbial production strains and bioprocess engineering. *Eng. Life Sci.* **17**: 1–22.

Liebl, W., Bayerl, A., Schein, B., Stillner, U., and Schleifer, K.H. (1989) High efficiency electroporation of intact *Corynebacterium glutamicum* cells. *FEMS Microbiol. Lett.* **53**: 299–303.

Meiswinkel, T.M., Gopinath, V., Lindner, S.N., Nampoothiri, K.M., and Wendisch, V.F. (2013) Accelerated pentose utilization by *Corynebacterium glutamicum* for accelerated production of lysine, glutamate, ornithine and putrescine. *Microb. Biotechnol.* **6**: 131–40.

van der Rest, M.E., Lange, C., and Molenaar, D. (1999) A heat shock following electroporation induces highly efficient transformation of *Corynebacterium glutamicum* with xenogeneic plasmid DNA. *Appl. Microbiol. Biotechnol.* **52**: 541–5.

Sambrook, J. and Russell, D.W. (2001) Molecular cloning: a laboratory manual 3rd ed. Cold Spring Harbor Laboratory Press, Cold Spring Harbor, NY, USA.

Schäfer, A., Tauch, A., Jäger, W., Kalinowski, J., Thierbach, G., and Pühler, A. (1994) Small mobilizable multi-purpose cloning vectors derived from the *Escherichia coli* plasmids pK18 and pK19: selection of defined deletions in the chromosome of *Corynebacterium glutamicum*. *Gene* **145**: 69–73.

Schneider, J., Niermann, K., and Wendisch, V.F. (2011) Production of the amino acids L-glutamate, L-lysine, L-ornithine and L-arginine from arabinose by recombinant *Corynebacterium glutamicum*. *J. Biotechnol.* **154**: 191–8.

Schreiner, M.E., Fiur, D., Holátko, J., Pátek, M., and Eikmanns, B.J. (2005) E1 enzyme of the pyruvate dehydrogenase complex in *Corynebacterium glutamicum*: molecular analysis of the gene and phylogenetic aspects. *J. Bacteriol.* **187**: 6005–18.

Shah, A., Eikmanns, B.J., Yukawa, H., Marin, K., Wendisch, V., Eikmanns, B., and Prieto, M. (2016) Transcriptional regulation of the β-type carbonic anhydrase gene *bca* by RamA in *Corynebacterium glutamicum*. *PLoS One* **11**: e0154382.

Tauch, A., Kirchner, O., Löffler, B., Götker, S., Pühler, A., and Kalinowski, J. (2002) Efficient electrotransformation of *Corynebacterium diphtheriae* with a mini-replicon derived from the *Corynebacterium glutamicum* plasmid pGA1. *Curr. Microbiol.* **45**: 362–7.

Unthan, S., Baumgart, M., Radek, A., Herbst, M., Siebert, D., Brühl, N., et al. (2014) Chassis organism from *Corynebacterium glutamicum* – a top-down approach to identify and delete irrelevant gene clusters. *Biotechnol. J.* **10**: 290–301.

1. aInstitute of Biochemical Engineering, University of Stuttgart, D-70569 Stuttgart, Germany

   Corresponding author: Dr. Bastian Blombach (blombach@ibvt.uni-stuttgart.de), Institute of Biochemical Engineering, University of Stuttgart, Allmandring 31, D-70569 Stuttgart, Germany. [↑](#footnote-ref-1)
